# Supplementary material for: Effects of Loganin on Bone Formation and Resorption In Vitro and In Vivo
Source: Int J Mol Sci. 2022 Nov 16;23(22):14128. doi: 10.3390/ijms232214128 (PMC9696919; doi:10.3390/ijms232214128)
Supplement: Supplementary file 1 [file ijms-23-14128-s001.zip › ijms-2011501-supplementary.pdf]

Effects of Loganin on Bone Formation and Resorption In Vitro and In Vivo

Chang-Gun Lee, Do-Wan Kim, Jeonghyun Kim, Laxmi Prasad Uprety, Kang-Il Oh, Shivani Singh, Jisu Yoo, Hyun-Seok Jin, Tae Hyun Choi, Eunkuk Park and Seon-Yong Jeong

Supplementary Table S1. Gene specific primers used in this study.

Gene	Primer sequence
Alpl	Forward: 5′-TCC CAC GTT TTC ACA TTC GG-3′<br>Reverse: 5′-CCC GTT ACC ATA TAG GAT AGC C-3′
Runx2	Forward: 5′-TAA AGT GAC AGT GGA CGG TCC C-3′<br>Reverse: 5′-AAT GCG CCC TAA ATC ACT GAG G-3′
Bglap	Forward: 5′-TAG TGA ACA GAC TCC GGC GCT A-3′<br>Reverse: 5′-TGT AGG CGG TCT TCA AGC CAT-3′
Ctsk	Forward: 5′-AAT ACC TCC CTC TCG ATC CTA CA-3′<br>Reverse: 5′-TGG TTC TTG ACT GGA GTA ACG TA-3′
Acp5	Forward: 5′-TGG TAT GTG CTG GCT GGA AAC-3′<br>Reverse: 5′-GCA GAG GCA TAC TTG TAC CG-3′
Mmp9	Forward: 5′-GCA GAG GCA TAC TTG TAC CG-3′<br>Reverse: 5′-TGA TGT TAT GAT GGT CCC ACT TG-3′
Gapdh	Forward: 5′-AGG TCG GTG TGA ACG GAT TTG-3′<br>Reverse: 5′-TGT AGA CCA TGT AGT TGA GGT CA-3′
Hprt	Forward: 5′-GAG GAG TCC TGT TGA TGT TGC CAG-3′<br>Reverse: 5′-GGC TGG CCT ATA GGC TCA TAG TGC-3′
